# The intersection of DNA replication with antisense 3′ RNA processing in *Arabidopsis FLC* chromatin silencing

**DOI:** 10.1073/pnas.2107483118

**Published:** 2021-07-06

**Authors:** Colette L. Baxter, Saša Šviković, Julian E. Sale, Caroline Dean, Silvia Costa

**Affiliations:** ^a^Cell and Developmental Biology Department, John Innes Centre, Norwich NR4 7UH, United Kingdom;; ^b^Division of Protein & Nucleic Acid Chemistry, MRC Laboratory of Molecular Biology, Cambridge CB2 0QH, United Kingdom

**Keywords:** DNA replication, transcription, chromatin silencing, *Arabidopsis*, chicken DT40

## Abstract

How noncoding transcription influences chromatin states is still unclear. The *Arabidopsis* floral repressor gene *FLC* is quantitatively regulated through an antisense-mediated chromatin silencing mechanism. The *FLC* antisense transcripts form a cotranscriptional R-loop that is dynamically resolved by RNA 3′ processing factors (FCA and FY), and this is linked to chromatin silencing. Here, we investigate this silencing mechanism and show, using single-molecule DNA fiber analysis, that FCA and FY are required for unimpeded replication fork progression across the *Arabidopsis* genome. We then employ the chicken DT40 cell line system, developed to investigate sequence-dependent replication and chromatin inheritance, and find that *FLC* R-loop sequences have an orientation-dependent ability to stall replication forks. These data suggest a coordination between RNA 3′ processing of antisense RNA and replication fork progression in the inheritance of chromatin silencing at *FLC*.

Genetic analysis identified FCA (an RNA binding protein) and FY (a component of the cleavage polyadenylation specificity factor complex) as components of an antisense-mediated chromatin silencing mechanism at *Arabidopsis FLC* (1). Their 3′ processing activities have widespread roles in transcriptional termination in the *Arabidopsis* genome (2). At *FLC*, they resolve an NDX-stabilized R-loop generating a proximally polyadenylated *COOLAIR* transcript (3–6). Resolution of the R-loop recruits chromatin modifiers, including the H3K4me1 demethylase FLD, required for low *FLC* expression (7). FLD interacts with the SET domain protein SDG26, which, in turn, transiently interacts with FY, providing a physical link between R-loop resolution and chromatin silencing at *FLC* (4). These activities are part of the autonomous floral pathway, which promotes rapid flowering in accessions such as Columbia, and are completely independent of the cold-induced Polycomb silencing mechanism in the vernalization process. The connections between R-loop resolution and chromatin inheritance at *FLC* remain poorly understood.

Recent work in mammalian cells has shown that RNA 3′ processing is linked to DNA replication (8). Cotranscriptional R-loops have also been implicated in impeding replication fork progression to impact epigenetic inheritance (9). Replication/transcription collisions have been proposed to control chromatin states (10). We, therefore, set out to test whether FY- and FCA-mediated RNA 3′ processing interferes with ongoing *Arabidopsis* replication forks. We developed a single-molecule DNA combing assay for *Arabidopsis*, based on the in vivo incorporation in seedlings of the thymidine analog CldU and the stretching of individual DNA fibers onto glass slides (*SI Appendix*). Progression of replication forks was tested at the genomic level in wild-type (WT) Col-0, *fy-2*, and *fca-9 Arabidopsis* mutant seedlings. A statistically significant reduction in the overall length of pairs of replication forks was observed in *fy-2* compared to Col-0 and *fca-9* (Fig. 1*A*). Closer examination of individual fork pairs revealed that, in Col-0, most replication forks were symmetric, with the left and right forks departing from the same origin having the same length of replicated DNA, indicating they both progress unimpeded. In contrast, in *fy-2* and, to a lesser extent, in *fca-9*, replication forks were found to be asymmetric, suggesting that one of the diverging forks is being stalled (Fig. 1 *B* and *C*). The lower frequency of asymmetric forks in *fca-9* compared to that in *fy-2* is likely to be explained by the fact that FCA appears to function at only a subset of FY targets (2). These results indicate that defects in *Arabidopsis* 3′ RNA processing impact replication fork progression.

**Fig. 1.**
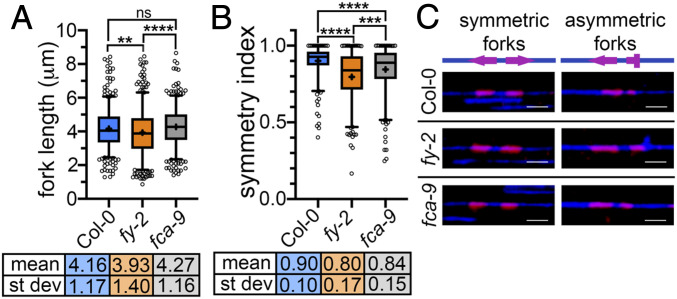
The 3′ end RNA processing mediated by FY and FCA is required for unimpeded replication fork progression across the *Arabidopsis* genome. (*A*) Analysis of the length of replication fork pairs by DNA combing; scored 480 replication tracks per genotype; *n* = 2. (*B*) Replication fork symmetry analysis, symmetry index = 1 when the left and right forks within a fork pair are of equal length; analyzed 240 fork pairs per genotype; *n* = 2. For *A* and *B*, *n* = number of biological replicates; the graph’s whiskers span the 5th to 95th percentiles, and dots represent the outliers; *P* value: ** ≤ 0.005, *** ≤ 0.0005, **** ≤ 0.0001, ns = not significant, one-way ANOVA with Dunnett’s multiple comparison test. (*C*) Representative images of two pairs of replication forks, one where the left and right fork have the same length (symmetric forks), and one where they have different lengths (asymmetric forks); replicated DNA (magenta), unreplicated DNA (blue), and schematic representation of the forks onto the DNA fiber. (Scale bars, 5 μm.)

We then wanted to understand whether FCA and FY processing of the *FLC* R-loop influences replication fork progression. This is technically not feasible *in planta*, so we exploited the chicken DT40 cell line, which provides a well-characterized system to assay the effect of heterologous sequences on progression of the replisome (9, 11). Sequences are cloned downstream of the chicken *BU-1* transcription start site, and their ability to confer expression instability to *BU-1* expression is monitored by fluctuation analysis. Such instability manifests when sequences cloned onto the leading strand form secondary structures that significantly stall the replisome, causing defective histone redeposition and the consequent loss of epigenetic information. Sequences cloned onto the lagging strand, instead, have been shown not to cause instability (9, 11, 12). To test whether the *FLC* 3′ sequences influence replisome progression, we cloned an 815-bp *FLC* fragment, which corresponds to the R-loop−forming sequences, into the *BU-1* locus, in both orientations (Fig. 2*A*). As experimental controls, we used cells containing a previously characterized poly-purine (GAA)_10_ repeat at the same locus, which forms a triplex DNA structure and an R-loop (9), or cells without any predicted structure at the *BU-1* locus (WT ∆G4). Similar to the (GAA)_10_ repeat construct, the *FLC* sequences caused no significant changes in *BU-1* expression stability in WT cells (Fig. 2*B*). However, replisome stalling may be masked by mechanisms that restart DNA replication, such as the primase-polymerase PrimPol, which reprimes replication downstream of fork stalling structures; PrimPol loss increases the probability of a leading strand stall leading to disruption of the *BU-1* epigenetic state (9, 11). Therefore, to sensitize the system, we tested a *primpol* mutant line and found a modest, but not statistically significant, increase in *BU-1* instability when the *FLC* coding strand undergoes leading strand replication. However, in the orientation where the *FLC* template strand undergoes leading strand replication, *BU-1* instability was pronounced (Fig. 2*B*). Structure predictions of the *FLC* 3′ sequences suggest possible triplex-forming and G-quadruplex−forming regions (see *SI Appendix*). Furthermore, the *FLC* sequences remain capable of forming R-loops, as shown by RNase H-sensitive S9.6 antibody immunoprecipitation (Fig. 2*C*), and R-loop formation contributes to *BU-1* instability in *primpol* cells overexpressing RNase H1 (Fig. 2*D*). The use of the DT40 system thus identified sequences on the template strand of *FLC* that effectively stall the replisome in an R-loop−dependent manner.

**Fig. 2.**
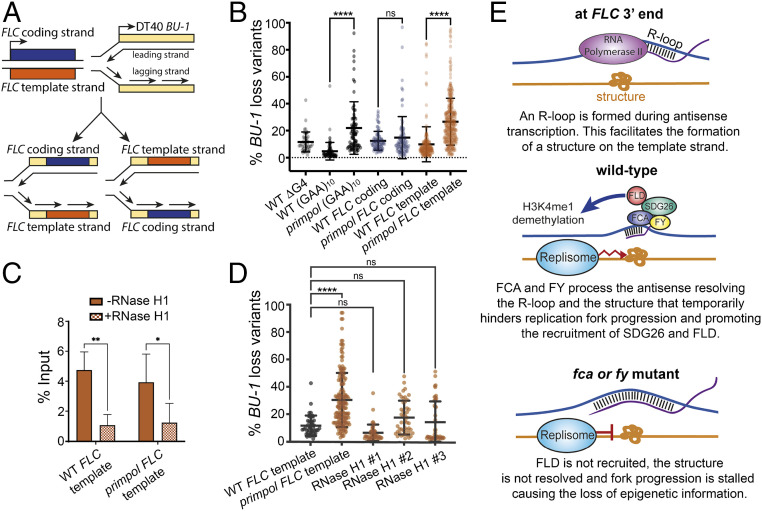
An R-loop coupled DNA structure stalls the replisome. (*A*) Schematic illustrating the cloning of the *FLC* 3′ end sequences into the chicken DT40 *BU-1* locus in both orientations to create cell lines with the *FLC* coding or template strand undergoing leading strand synthesis. (*B*) Fluctuation analysis of *BU-1* expression in WT and *primpol* mutant cell lines with the WT G-quadruplex deleted (WT ∆G4) or replaced with the following sequences: with a GAA_10_ repeat (GAA)_10_ or the *FLC* 3′ end region in both orientations (*FLC* coding and *FLC* template). Points represent the percentage of cells with *BU-1* expression loss in each individual clone tested; *n* > 2. (*C*) DNA:RNA immunoprecipitation (DRIP)-qPCR signal across the *FLC* cloned sequence represented as a percentage of the input sample; *n* = 5. (*D*) Fluctuation analysis of *BU-1* expression in three *primpol FLC* template cell lines overexpressing RNaseH1. Points represent the percentage of cells with *BU-1* expression loss in each individual clone tested; *n* > 2; see *SI Appendix* for details; plots show mean ± SD; *P* value: * ≤ 0.05, ** ≤ 0.005, **** ≤ 0.0001, one-way (*B* and *D*) and two-way (*C*) ANOVA with Sidak multiple comparison test. (*E*) Schematic illustrating the model whereby FCA and FY efficiently 3′ process the *COOLAIR*-generated R-loop and destabilize the structure on the single-stranded template DNA. This slows replication fork progression, providing a temporal window for the propagation of chromatin silencing at the *FLC* locus.

Together, our data allow us to propose a model whereby, *in planta*, FCA and FY recognize an NDX-stabilized R-loop and efficiently terminate the *COOLAIR* transcript to resolve the R-loop. These R-loop dynamics would influence formation of a replisome-stalling structure on the single-stranded DNA strand opposite the R-loop. In *fca* or *fy* mutants, where the R-loop is not resolved, the structure would form and stall the replication fork, and SDG26, FLD-mediated chromatin silencing would fail to be propagated (Fig. 2*E*). In WT cells, we envisage that the R-loop dynamics cause replisome slowing, but not stalling, creating a temporal window that facilitates recruitment and activity of FLD (Fig. 2*E*). Epigenetic silencing mediated by LSD1, the homolog of FLD, has been linked to R-loop homeostasis in mammalian cells (13). LSD1 is also involved in replication fork pausing at the mating-type locus in fission yeast (14), and the Rik1 complex, which associates with the replisome, silences heterochromatin via the H3K4me3 demethylase Lid2 (10). These and our findings suggest the importance of the interplay between RNA 3′ processing, R-loop stabilization, and replication fork progression in chromatin inheritance.

## Materials and Methods

Detailed descriptions are provided as *SI Appendix*.

For the *Arabidopsis* DNA replication/combing assay, seedlings were in vivo labeled with CldU, nuclei were extracted and embedded in agarose blocks, DNA was deproteinized, and the DNA fibers were stretched onto glass coverslips. Incorporated CldU and DNA fibers were immunodetected, and images were analyzed using Fiji software.

For the DT40 cell culture replication assay, the 3′ *FLC* region was inserted into the *BU-1* locus by cloning the region into the *BU-1* targeting vector, and transfecting into DT40 cells; successful insertion was determined by puromycin selection and flow cytometry. Overexpression of RNaseH1 was achieved by viral transduction of chicken RNaseH1 and RNaseH1 expression established by flow cytometry. For *BU-1* fluctuation analyses, cells were grown for 20 generations and stained with anti-*BU*-1a antibodies, and clones were analyzed by flow cytometry for the percentage of cells that had lost expression.

## Supplementary Material

Supplementary File

## Data Availability

All study data are included in the article and *SI Appendix*.
