# Supplementary material for: The intersection of DNA replication with antisense 3′ RNA processing in Arabidopsis FLC chromatin silencing
Source: Proc Natl Acad Sci U S A. 2021 Jul 6;118(28):e2107483118. doi: 10.1073/pnas.2107483118 (PMC8285979; doi:10.1073/pnas.2107483118)
Supplement: Supplementary File [file pnas.2107483118.sapp.pdf]

PNAS
www.pnas.org

Supplementary Information for

The intersection of DNA replication with antisense 3’ RNA processing in Arabidopsis *FLC* chromatin silencing

Colette L. Baxter[a], Saša Šviković[b,c], Julian E. Sale[b]*, Caroline Dean[a]* and Silvia Costa[a]*

[a]John Innes Centre, Norwich, UK; [b] MRC Laboratory of Molecular Biology, Cambridge, UK; [c] Present address: AstraZeneca, Gothenburg, Sweden

*Corresponding authors: Julian Sale, Caroline Dean and Silvia Costa
Email: jes@mrc-lmb.cam.ac.uk; Caroline.Dean@jic.ac.uk; Silvia.Costa@jic.ac.uk;

This PDF file includes:

Extended Materials and Methods

Materials and Methods

Plant material and growth conditions

The *fca-9* mutant was described in (1) and the *fy-2* mutant in (2).
Seeds were surface sterilized in 30% v/v sodium hypochlorite for 15 min and rinsed 5 times in sterile distilled water.

Single molecule DNA combing

In vivo labelling: Col-0, *fy-2* and *fca-9* seedlings were grown in long day conditions (16 hours light and 8 hours darkness) for 7 days in liquid MS medium supplemented with 0.5% sucrose on a rotary shaker, the medium was gently removed and replaced with fresh MS medium supplemented with 40 μM CldU and the seedlings were labelled for 30 minutes. To block the labelling reaction the medium was quickly removed, seedlings were gently patted with tissue paper to remove excess liquid and then frozen in liquid nitrogen. The procedure was carried out rapidly and consistently by timing it to 80 seconds. All three genotypes were processed in parallel to ensure the data from the *in vivo* incorporation of CldU between the three genotypes could be compared.

Nuclei extraction: After labelling, the whole seedlings were ground in liquid nitrogen to a very fine powder. The powder was gradually and gently resuspended in nuclei extraction buffer (20 mM MOPS pH7, 20 mM NaCl, 90 mM KCl, 2 mM EDTA pH8, 0.5 M sucrose, 0.1% v/v 2-mercaptoethanol) using 10 ml of buffer for each gram of seedlings. The suspension was filtered through a double layer of Miracloth into conical tubes and spun at 1000 rcf. The pellet was resuspended into nuclei extraction buffer and loaded on the top of 2.5 M sucrose and 60% Percoll gradient and spun for 45 min at 1000 rcf. The upper phase was collected and diluted 1:4 with nuclei extraction buffer, mixed gently and spun at 1000 rcf for 10 min. The supernatant was removed, nuclei were washed twice in extraction buffer and resuspended.

Plugs preparation: The nuclei were resuspended in a small amount of nuclei extraction buffer and an equal volume of 1.4% low melting point agarose was added mixing gently. The nuclei solution was cast into plug molds (BioRad) and plugs were allowed to polymerize at 4ºC. To deproteinize the nuclei the plugs were incubated in proteinase K solution (0.5 M EDTA pH8, 1% sodium lauroylsarcosine, 1 mg/ml proteinase K) for 16 hours in a water bath at 50ºC, the proteinase K solution was then replaced with freshly made solution and incubated for an additional 8 hours. The proteinase K solution was removed, and plugs were washed three times in an excess of wash buffer (10 mM Tris-HCl pH 8 and 10 mM EDTA pH 8) for three hours each time under constant, but gentle agitation. Plugs were stored in buffer 5 from FiberPrep kit (Genomic Vision, Bagneux, France) and shipped to Genomic Vision, Bagneux France, for processing.

DNA combing and immunodetection: DNA combing and immunodetection were performed according to the EasyComb service procedures (Genomic Vision, Bagneux, France). Briefly, from the plugs containing the nuclei, single and long DNA molecules were extracted and stretched at a constant speed on specifically treated coverslips. After immunodetection of CldU and ssDNA, coverslips were scanned with FiberVision® scanner and images were analyzed.

Image and data analysis: Two intact CldU (magenta) replication tracks, which were no more than four microns apart and were flanked by counterstained ssDNA (blue) were classed as a fork pair. Only those tracks fulfilling these criteria were selected and their length was measured using Fiji software (https://imagej.nih.gov/ij/), a version of ImageJ.

We single-labelled our tracks with only CldU therefore we cannot discriminate between diverging replication forks, which derive from the same origin, and converging replication forks, which instead derive from separate origins. However, we minimized the presence of such converging events by keeping the labelling time to the minimum that allowed us to detect measurable tracks. The labelling time of 30 minutes we used produced relatively short tracks, when taking into consideration the length of the tracks obtained by other researchers using equivalent labelling times with mammalian cell cultures. The uptake of thymidine analogues by an intact multicellular living organism, like our seedlings, would be expected to take longer than the uptake by cells in cultures, hence the reduced length of tracks. However, the length of our replication tracks is consistent with that reported in the only previously published analysis of Arabidopsis DNA labelled fibers dating back to 1978, in which fibers were labelled with tritiated thymidine (3).

The symmetry index of a fork pair was calculated by dividing the shorter track by the longer track within each fork pair. Symmetry index =1 when the left and right tracks within a fork pair are of equal length and so they are symmetric, numbers lower than 1 point to asymmetric fork pairs.

In Fig.1, in the graphs the whiskers span the 5 to 95 percentiles, the dots represent the outliers, the box extends from the 25th to the 75th percentiles, the line in the middle of box represents the median and the cross represents the mean.

DT40 cell culture and mutants

The DT40 cell culture is described in (4), the WT ΔG4 cell line in (5), the *primpol* ΔG4 line in (6), the WT GAA_{10} and *primpol* (GAA_{10}) lines in (7). The 3' region of *FLC* was PCR amplified using the primers FLC forward CTATCAGGCGCGCCTGCTTCCAAACTTAAAAGCTTAAAC and FLC reverse TATTCTAGGCGCGCCCCTTCATGGATGACGGAACTACGG, which have an AscI site added, and digested with AscI. The construct was then cloned into the *BU-1* targeting construct using the MluI sites and screened for orientation by Sanger sequencing. The targeting constructs were then digested with NotI and transfected into WT ΔG4 and *primpol* ΔG4 cells by electroporation. Successfully transfected cells were selected by puromycin treatment and flow cytometry. The targeting cassette was removed by transfecting the cell with a Cre expressing vector and successful removal was screened for by flow cytometry as described (5).

Fluctuation analysis

BU-1 fluctuation analyses were performed as described previously in (5, 8) with some minor changes. In brief, confluent cells (0.4×10^6 to 2×10^6/ml) were plated into 96-well plates to obtain a single cell per well either by limiting dilutions or *BU-1* positive sorting on the MOFLO sorting cytometer. Cells were grown for 20 generations and then directly stained with anti-BU-1a conjugated with phycoerythrin (Invitrogen 21-1A4-PE MA5-28754) at 1:100 dilution for 10 min at 37°C. Cells were analyzed by flow cytometry using an LSR Fortessa cytometer (BD

Biosciences). At least two independent fluctuation analyses were performed with at least two cell lines derived from individual transfected clones per construct. Within each experiment 22–48 individual clones were analyzed for each cell line.

RNaseH overexpression line

Overexpression of RNaseH1 was achieved by transducing the DT40 cells. Briefly, the cDNA encoding for chicken RNaseH1 lacking mitochondrial localization sequence fused to YFP was subcloned from BML4-RNaseH1 ΔMLS-YFP plasmid (7) as a HindIII-NotI fragment into M6P-hisd (a γ-retroviral vector with IRES-driven L-histidinol resistance) (9). Viral particles were prepared by triple transfection of HEK293ET cells (grown in IMDM supplemented with 10% FBS and 1% antibiotics) with M6P-RNaseH1-YFP-hisd, pMD-OGP and pMD-VSV-G (in 1:0.7:0.3 weight ratios) with TransIT-LT1 (Mirus, MIR2300) as per manufacturer's instructions. DT40 cells were transduced with the viral supernatant at 0.1-0.3 MOI by transfecting 200,000 cells for 2 h at 700 g at 37°C in the presence of 8 μg/ml of polybrene (Merck Millipore, TR-1003). 48 hours post transduction, 0.5-4 cells were plated per well of a 96-well plate and allowed to expand into colonies by incubating for 10 days in the presence of 1 mg/ml L-histidinol dihydrochloride (Sigma H6647). RNaseH1-YFP expression was confirmed by flow cytometry.

DNA:RNA immunoprecipitation (DRIP)

DRIP was performed mostly as described in (7). In brief, 30 million DT40 cells were harvested by centrifugation, washed in PBS and lysed using hypotonic lysis buffer (10mM Tris-HCl pH 7.5, 10 mM NaCl, 2.5 mM $MgCl_2$) with 0.5% (vol/vol) NP-40. The nuclei were pelleted by centrifugation, resuspended in nuclei lysis buffer (25 mM Tris-HCl pH 7.5, 1% SDS, 5 mM EDTA) and treated with Proteinase K (Thermo Fischer) overnight. SDS and contaminating proteins were removed by adding 5 M KOAc pH 5.5 and centrifuging. DNA was precipitated from the supernatant with isopropanol. Samples were then split and treated with 40 μg RNaseA (Thermo Fischer, EN0531) alone or in combination with 20U RNaseH (NEB, M0297) overnight. The resulting DNA was then sonicated using the Bioruptor sonicator (Diganode) using 30 seconds on, 30 seconds off for 30 cycles on high setting, yielding an average fragment size of 1 kb. Input control fractions were taken and samples were subsequently diluted to 1 ml with IP dilution buffer (16.6 mM Tris–HCl pH 7.5, 1.2 mM EDTA, 165 mM NaCl, 1.1% Triton X-100, 0.01% SDS) and immunoprecipitated with 10 μg S9.6 antibody overnight at 4°C. Protein G Dynabeads (Thermo Fischer, 10003D) were then added to the samples, incubated for 1 hours at 4°C. The beads were then washed with low-salt, high-salt and LiCl wash buffers and TE buffer. The DNA:RNA hybrids were eluted for 2 h at 65°C in elution buffer (10 mM sodium phosphate buffer pH 7.0, 140 mM NaCl, 0.05% Triton X-100) and purified with PCR Purification Kit (QIAGEN). The samples were then analyzed by qPCR using the primers FLC forward CTGCTGGACAAATCTCCGA and FLC reverse GGATTTTGATTTCAACCGCCGA, the signal quantified as a percentage of the input signal.

DNA secondary structure prediction

G-quadruplexes were predicted using the online platform QGRS mapper (10) with a maximum length of 30 nucleotides, a minimum G-group of 2, and a loop size of 0 to 36. Triplexes were predicted using the Triplex Bioconductor package in R (11) which predicts triplexes based on the purine content.

SI References

1. F. Liu *et al.*, The Arabidopsis RNA-binding protein FCA requires a lysine-specific demethylase 1 homolog to downregulate *FLC*. *Molecular cell* **28**, 398-407 (2007).
2. I. R. Henderson, F. Liu, S. Drea, G. G. Simpson, C. Dean, An allelic series reveals essential roles for FY in plant development in addition to flowering-time control. *Development (Cambridge, England)* **132**, 3597-3607 (2005).
3. J. Van't Hof, A. Kuniyuki, C. A. Bjerknes, The size and number of replicon families of chromosomal DNA of *Arabidopsis thaliana*. *Chromosoma* **68**, 269-285 (1978).
4. L. J. Simpson, J. E. Sale, Rev1 is essential for DNA damage tolerance and non-templated immunoglobulin gene mutation in a vertebrate cell line. *The EMBO journal* **22**, 1654-1664 (2003).
5. D. Schiavone *et al.*, Determinants of G quadruplex-induced epigenetic instability in REV1-deficient cells. *The EMBO journal* **33**, 2507-2520 (2014).
6. D. Schiavone *et al.*, PrimPol is required for replicative tolerance of G quadruplexes in vertebrate cells. *Molecular cell* **61**, 161-169 (2016).
7. S. Šviković *et al.*, R-loop formation during S phase is restricted by PrimPol-mediated repriming. *The EMBO journal* **38** (2019).
8. G. Guilbaud *et al.*, Local epigenetic reprogramming induced by G-quadruplex ligands. *Nat Chem* **9**, 1110-1117 (2017).
9. S. Rahighi *et al.*, Specific recognition of linear ubiquitin chains by NEMO is important for NF-kappaB activation. *Cell* **136**, 1098-1109 (2009).
10. O. Kikin, L. D'Antonio, P. S. Bagga, QGRS Mapper: a web-based server for predicting G-quadruplexes in nucleotide sequences. *Nucleic acids research* **34**, W676-682 (2006).
11. J. Hon, T. Martínek, K. Rajdl, M. Lexa, Triplex: an R/Bioconductor package for identification and visualization of potential intramolecular triplex patterns in DNA sequences. *Bioinformatics* **29**, 1900-1901 (2013).
